# Performance and microbiota of the digestive tract of Nellore calves supplemented with fungi isolated from bovine rumen

**DOI:** 10.14202/vetworld.2021.2686-2693

**Published:** 2021-10-21

**Authors:** Thiago Alves Xavier dos Santos, Luís Miguel Gonçalves Fernandes, Pedro Paulo Xavier Carvalho, Valdo Soares Martins Júnior, Suze Adriane Fonseca, Amalia Saturnino Chaves, Eduardo Robson Duarte

**Affiliations:** 1 Institute of Agricultural Science, Universidade Federal de Minas Gerais, Campus Montes Claros - MG, 39404-547, Brazil; 2 Department of Veterinary Medicine, Universidade Federal de Juiz de Fora, Juiz de Fora - MG, 36036-900, Brazil.

**Keywords:** Enterobacteriaceae, ruminal microbiota, rumen protozoa, semiarid region, weaning

## Abstract

**Background and Aim:**

In tropical semiarid regions, supplementation with fungi could contribute to rumen modulation, promoting greater production of fibrolytic enzymes and degradation of forage. The objective of this study was to analyze the effect of supplementation with fungi, isolated from the bovine rumen, on the performance and microbiota of the digestive tract of Nellore calves.

**Materials and Methods:**

The experiment was conducted in randomized blocks evaluating eight Nellore calves that were daily supplemented with isolates of *Aspergillus terreus* and *Trichoderma longibrachiatum*, along with eight calves that were not supplemented. After 55 days, the animals were weighed, and samples of rumen fluid and feces were collected for analysis. The characteristics that showed normal distribution were subjected to analysis of variance and compared using Tukey’s test. Whereas, the variables that did not show normal distribution were subjected to the Kruskal–Wallis test, and the frequencies of the bacterial and fungal genera were compared using the Chi-square test.

**Results:**

Supplementation with fungi promoted the reduction in ruminal pH (p<0.05). However, the final live weight; average daily weight gain; total weight gain; rumen protozoa; and the count of Enterobacteriaceae, mycelial fungi, and yeasts of ruminal fluid and feces were not influenced by supplementation (p>0.05). Moreover, the protozoa *Eodinium* spp. was identified only in supplemented calves (p<0.05).

**Conclusion:**

Supplementation with the fungi presented the potential for use as possible additives because it did not alter the physiological parameters of the facultative anaerobic microbiota composition in the rumen and feces. In addition, it favored the presence of the ciliate genus *Eodinium*. However, further studies should be performed to better define suitable dosages for supplementation.

## Introduction

Cattle directly depend on their gastrointestinal microbiota for energy metabolism, the digestion of nutrients, and the reduction of pathogenic microorganisms [1]. In particular, the stress levels and health risks of calves are high after weaning because they are still very susceptible to diseases and digestive changes during this period [2,3]. To reduce risks and improve food efficiency, the prophylactic administration of antimicrobials has been promoted [4]. In this context, anaerobic fungi from the rumen may play an important role as a microbial additive in ruminant supplementation due to its ability to degrade forages by producing enzymes with fibrolytic activity, which are capable of increasing the availability of soluble carbohydrates and amino acids [5]. This action favors the growth of rumen microbiota, thereby providing improvements in the ecosystem, in ruminal parameters, and in the productivity of the animals [6,7]. However, these fungi show laborious growth in laboratory conditions, which makes their use on an industrial scale difficult.

The anaerobic facultative fungi may be present in the rumen environment in proportions of approximately 1×10^4^ Colony-Forming Units (CFU)/mL, in animals raised on tropical pastures in the dry season [8,9]. These fungal isolates have been found to produce cellulases, which are enzymes important for the solubilization of fibers in tropical forages [10,11] superiorly. In a previous study, beef cattle fed on lignified tropical forage presented the genus *Aspergillus* and *Trichoderma* in ruminal fluid [9], which are fungi that have shown potential for animal supplementation and for biotechnological purposes [12,13]. Selected isolates of *Aspergillus* spp. from the rumen environment have been found to present high levels of enzyme production that solubilize the lignified cell walls of plants and they do not produce mycotoxins [9,14].

Therefore, in semiarid tropical regions, supplementation with fungi from the rumen could be an important alternative, contributing to improvements in the cellulolytic microbiota of the rumen and reducing the populations of pathogenic microorganisms. The objective of this study was to analyze the effects of supplementation with selected fungi isolated from the rumen, on the performance and microbiota of the digestive tract of Nellore calves.

## Materials and Methods

### Ethical approval

All procedures adopted by this study were approved by the Ethics Committee on the Use of Animals of the Federal University of Minas Gerais (UFMG), under number 209/2018.

### Study period and location

The study was carried out from September to November 2018. The study was conducted in Montes Claros city, located north of the state of Minas Gerais, Brazil (16°42′16′′ latitude south, 43°49′13′′ longitude west, and 646 m in altitude). The region’s climate is of the AW type, according to the Köppen classification, which is characterized by a rainy season in the summer and a dry season in the winter, with typical savanna vegetation. The average annual temperature is 24.2°C and the average annual rainfall is approximately 1050 mm, with the drought period being from April to October [15].

### Study design

The experiment was performed in a randomized block design containing 16 Nellore calves, approximately 8 months old. These comprised eight unregistered males and eight females with initial body weight (IBW) of 284.5 kg±38 kg. The diet was formulated according to the *National Research Council* (NRC) [16], for an average daily gain of 400 g, and consisted of 56.79% *Urochloa brizantha* hay and 43.21% concentrate (Table-1). This feed mixture was provided daily at 8:00 am and 3:00 pm. The quantities of food offered were adjusted daily according to the amount of leftovers, which were kept at 5% to allow consumption at will and water was provided *ad libitum*.

**Table-1:** Nutritional composition of the experimental diet.

Ingredients	Experimental diet
*Urochloa brizantha hay*	56.79
Groundcorn	28.73
Soy bean meal	2.23
Mineral core^1^	12.25
Composition	
Dry matter	93.00
Crude protein	10.96
Ethereal extract	1.17
Neutral detergent fiber	74.23
Acid detergent fiber	44.21
Mineral content	1.17

^1^Connan Pasto Seco®=Calcium (minimum/maximum) 20-90 g; Phosphorus (minimum) 15 g; Sodium (minimum) 50 g; Magnesium (minimum) 5.5 g; Sulfur (minimum) 9 g; Fluorine (maximum) 150 mg; Zinc (minimum) 610 mg; Copper (minimum) 200 mg; Manganese (minimum) 150 mg; Cobalt (minimum) 13 mg; Iodine (minimum) 45 mg; Selenium (minimum) 4.5 mg; Crude Protein (minimum) 350 g; NNP Equiv. PB (minimum) 295 g.

Samples of the diets were analyzed for dry matter content at 105ºC. Mineral matter, ether extract, and crude protein were analyzed according to the *Association of Official Analytical Chemists* [17]; whereas, neutral detergent fiber and acid detergent fiber were analyzed according to the methodology suggested by Van Soest *et al*. [18].

### Evaluation of selected fungi

The isolated fungi were from the rumen of Nellore steers raised in an extensive system on a *U. decumbens* pasture, with mineral supplementation containing urea [9]. These fungi were identified by the sequencing of the ribosomal DNA (rDNA), which was obtained from the amplification of the ITS region of the rDNA using the ITS1 (TCCGTAGGTGAACCTGCGG) and ITS4 (TCCTCCGCTTATTGATATGC) primers [19]. The products were analyzed in DYEnamic (Amersham Biosciences, USA) using the automated sequencing system MegaBYTE 1000 (Genome Analysis Center and Gene Expression). The obtained sequences were analyzed using BLASTn v. 2.215 of BLAST 2.0 [19]. These species were considered to be isolates with similarities of 99% or more, and the sequences were deposited in the GenBank as *Aspergillus terreus* (KF781532) and *Trichoderma longibrachiatum* (KF781535).

These isolates were selected because they are present in greater proportions in the digestive tract of cattle, do not produce mycotoxins, have no reports of causing mycoses, and show higher production levels of fibrolytic enzymes when compared to other enzymes in the digestive tract [10,11].

### Experimental groups

One group with eight calves (SC) supplemented with a mixture of the fungi, *A. terreus* (isolated VN15) and *T. longibrachiatum* (isolated VN20), was compared to the group of control calves (CC). This control group was also composed of eight calves that received only the culture medium for these microorganisms. Each group comprised four male and four female calves for block study.

The experiment lasted 70 days, with 15 days of adaptation to the diet and pens, as well as vaccination against coccidiosis and the use of anthelmintics (Albendazole 15 mg/kg, subcutaneous). The other 55 days were for supplying supplementation with fungi and the experimental diet. The animals were confined in individual pens that were covered in the trough area, with dimensions of 1.5 m (width)×3.0 m (length), and were parallel to each other.

The fungus culture medium was composed of 2.5 g soybean, 2.5 g starch, 2.5 g dextrose, and 2% *U. decumbens* hay for 1 L of distilled water, as described by Freitas [20]. Before the first feeding, the SC were daily supplemented with 160 mL of culture medium containing 4.4×10^8^ CFU/mL of *A. terreus* and 2.0×10^8^ CFU/mL of *T. longibrachiatum*, mixed with 200 g of concentrate. The CC received the same amount of the culture medium without the fungi in the concentrate.

### Sample collection

The animals were weighed every 15 days of the experimental period with a mechanical balance (FIZIOLA^®^, model 3106000, São Paulo, Brazil). The initial and final weighing was done after fasting solids for approximately 16 h before feeding to minimize the differences in filling between the animals.

After 55 days of experimentation, ruminal fluid and feces were collected at 7:00 am-11:00 am, and the calves were immobilized in a containment. The ruminal fluid was collected at the ventral part of the left abdomen, cranially below the knee joint, with approximately 5 cm^2^, after performing the trichotomy and asepsis with a solution of polyvinylpyrrolidone-iodine (PVP-iodine 1%). Approximately 15 mL of fluid was collected with sterile catheters and syringes. Before the collection of feces, asepsis of the perianal region was performed with the same PVP-iodine 1% antiseptic, and samples of approximately 100 g of feces were collected directly from the rectal ampoule using gloves and sterile plastic bags. All samples were stored for up to 1 h and transported in isothermal boxes containing recyclable ice [9].

### Physical-chemical characterization of ruminal fluid

In 5 mL subsamples of ruminal fluid, color, odor, and viscosity were evaluated. The microbial activity was estimated by the 0.03% methylene blue reduction test (PRAM) and the hydrogen potential (pH) was determined with a digital potentiometer (GEHAKA^®^, São Paulo, Brazil) [21].

### Analysis of aerobic and anaerobic microorganisms

For the microbial culture, feces and ruminal fluid were handled in a laminar flow hood to facilitate decimal dilutions in tubes containing sterile saline. Subsequently, aliquots of decimal dilutions were inoculated into sterile Petri dishes containing MacConkey agar medium (KASVI^®^, Terámo, Italy) and dilutions of 10^3^, 10^5^, and 10^7^ in plates containing Sabouraud agar (ACUMEDIA^®^, Michigan, United States). The plates were incubated at 37°C in a BOD stove and monitored for growth of Enterobacteriaceae for 48 h and up to 7 days for fungi [22]. The CFU/mL or CFU/g was quantified with the help of a colony counter.

The identification of the most frequent Enterobacteriaceae genera occurred after re-isolation and growth on plates containing MacConkey agar in an oven at 37°C for 24 h. After exponential growth, each isolate was inoculated into tubes containing Rugai and Araújo medium, modified by Pessoa and Silva (MBiolog Diagnósticos, Brazil). This classification considered the production capacity of indole, sulfides, and gases, in addition to the use of tryptophan, lysine, glucose, sucrose, urea, and motility [22,23].

For the identification of the genera of mycelial fungi, microcultures of 23 isolates from the ruminal fluid and feces of the calves were analyzed. The micromorphological characteristics were observed using an optical microscope in the 10 and 40× objectives, considering the characteristics described by Lacaz *et al*. [24], and Germain and Summerbel [25].

For rumen protozoan analyses, 100 μL samples of ruminal fluids were diluted in 900 μL of 10% formaldehyde solution to conserve the morphological structures of these eukaryotes [26]. For quantification, decimal dilutions were made in sterile saline, classifying small (40×60 μm), medium (100×150 μm), and large (100×150 μm) protozoa in a Sedgewick Rafter chamber (S52 glass, Pyser-SGI, Edenbridge, Kent, UK). This was done under an optical microscope with a 10× objective [21]. In the identification, aliquots of the 10^-1^ dilutions were evaluated, together with a drop of lugol, on microscopic slides for visualization of the microstructures of the protozoa, under an optical microscope [27]. The 40× objective was used to analyze approximately 700 protozoa per animal, which were classified and identified according to the key described in Dehority [26]. All procedures were performed in triplicate.

### Statistical analysis

The daily weight gain (DWG) was calculated from the slope coefficient of the straight line, resulting from the regression of individual body weight measurements, without fasting, as a function of time. This was done using the SAS REG procedure (2004). The performance characteristics and ruminal pH, which showed normal distribution, were analyzed using the Shapiro–Wilk test, by a model that included the effects of sex as a block and IBW as a covariate. The averages were compared by Tukey’s test at 5% probability, using the PROC GLM from SAS (2004).

For the analysis of microbial populations, the concentrations were transformed into log_10_ (x+10). The reduction in methylene blue (PRAM); ruminal and fecal values of Enterobacteriaceae, mycelial fungi, and yeasts; and ruminal protozoa that did not show normal distribution, were subjected to the Kruskal–Wallis test at 5% probability. The frequencies of the bacterial and fungal genera were compared by the Chi-square test (p=0.01), using PROC NPAR1WAY from SAS (2004).

## Results

### Analysis of performance, physicochemical characteristics, and macroscopic characteristics of ruminal fluid

The SC and CC groups did not show significant differences in the DWG, final body weight (FBW), and total weight gain (TWG), as shown in Table-2.

**Table-2:** Means and standard deviations (SD) of daily weight gain (DWG), total weight gain (TWG), final body weight (FBW), reduction in methylene blue (PRAM), and hydrogen potential (pH) of Nellore calves supplemented (SC) or not supplemented (CC) with ruminal fungi.

Variables	CC	SC^1^	SD	p-value
Performance				
DWG (kg/day)	0.44	0.48	0.19	0.25ª
TWG (kg)	24.00	26.63	10.82	0.26ª
FBW (kg)	294.38	275.13	38.00	0.79ª
Ruminal Fluid				
PRAM	6 min and 35 s	3 min and 31 s	3 min and 27 s	0.14^b^
pH	7.43	7.15	0.29	*0.02^a^

Min=Minutes. Sec=Seconds.

a Test of comparison of means by the Tukey test with 5% of significance.

b Test of comparison of means by the Kruskal–Wallis test with 5% significance. ^1^Supplementation with fungi: *Aspergillus terreus* (isolated VN15) and *Trichoderma longibrachiatum* (isolated VN20)

The characteristics of the color, odor, and viscosity of the ruminal fluid were not significantly influenced by fungal inoculation (p>0.05). The activity of the ruminal microbiota was high, with the PRAM being between 3 and 6 min; however, it too was uninfluenced by supplementation (p>0.05 – Table-2). The ruminal fluid pH was influenced by supplementation, being lower in SC when compared to CC (p>0.05 – Table-2).

### Quantification and identification of Enterobacteriaceae

The presence of Gram-negative rods that are facultative anaerobes, such as those of the Enterobacteriaceae family, was verified in all samples of ruminal fluid and feces from the evaluated calves. The average concentrations of these bacteria were not influenced by supplementation in SC (p>0.05 – Table-3). Similarly, regarding lactose-fermenting Enterobacteriaceae (Lac+) and non-lactose-fermenting Enterobacteriaceae (Lac-), the mean concentrations were not influenced by supplementation (p>0.05 – Table-3).

**Table-3:** Means of colony-forming units (CFU) and standard deviations (SD) of Enterobacteriaceae, mycelial fungi, and yeasts isolated from the ruminal fluid and feces of Nellore calves supplemented (SC) or not supplemented (CC) with ruminal fungi.

Variables	CC	SC^1^	SD	p-value
Ruminal Fluid (CFU/mL)				
Enterobacteriaceae	8.9×10^5^	1.2×10^5^	1.2×10^6^	0.20
Lac +	7.4×10^5^	8.6×10^4^	1.0×10^6^	0.17
Lac -	1.5×10^5^	3.2×10^4^	1.9×10^5^	0.15
Mycelial Fungi	1.5×10^4^	7.6×10^3^	2.0×10^4^	0.75
Yeasts	5.8×10^4^	5.5×10^4^	1.1×10^5^	0.87
Feces (CFU/g)				
Enterobacteriaceae	1.4×10^6^	9.0×10^5^	1.7×10^6^	0.52
Lac +	1.2×10^6^	8.8×10^5^	1.6×10^6^	0.59
Lac -	1.7×10^5^	1.8×10^4^	2.2×10^5^	0.28
Mycelial Fungi	2.6×10^6^	3.2×10^5^	4.2×10^6^	0.99
Yeasts	1.1×10^6^	2.1×10^7^	2.8×10^7^	0.83

Test of comparison of means by the Kruskal–Wallis test with 5% significance. Lac+=Lactose fermenting Enterobacteriaceae. Lac-=non-lactose-fermenting Enterobacteriaceae. ^1^Supplementation with fungi: *Aspergillus terreus* (isolated VN15) and *Trichoderma longibrachiatum* (isolated VN20)

Presumptively, 32 colonies of Enterobacteriaceae were identified. Furthermore, it was observed that the sites, ruminal fluid, and feces yielded no significant differences (p>0.05) in the genera of these bacteria, between the groups of calves evaluated (Table-4). From evaluating the frequencies of the genus, *Escherichia*, the distribution frequency was found to be similar to that of *Enterobacter* spp. (p=0.01), and was more frequent than the *Proteus* spp. and *Providencia* spp. (Table-4).

**Table-4:** Distribution of Enterobacteriaceae genera isolated from ruminal fluid and feces of Nellore calves supplemented (SC) or not supplemented (CC) with ruminal fungi.

Genera	Total (n)	Ruminal fluid				Feces			
	
		CC		SC^1^		CC		SC^1^	
			
		n	%	n	%	n	%	n	%
*Escherichia* spp.	15**	1	7	3	20	5	33	6	40
*Enterobacter* spp.	10**	5	50	3	30	1	10	1	10
*Proteus* spp.	5	1	20	2	40	1	20	1	20
*Providencia* spp.	2	1	50	-	-	1	50	-	-
Total	32	8		8		8		8	

** Values significantly higher when compared between treatments and between genders using the Chi-square test (p=0.01). ^1^Supplementation with fungi: *Aspergillus terreus* (isolated VN15) and *Trichoderma longibrachiatum* (isolated VN20)

### Quantification and identification of facultative anaerobic fungi

The average facultative anaerobic fungi in the ruminal fluid (6.2×10^4^±1.1×10^5^ CFU/mL) and in the feces (2.1×10^7^±3.9×10^7^ CFU/g) were similar between SC and CC groups (Table-3).

The genus of mycelial fungi that were most frequently identified in the samples of ruminal fluid and feces from calves was *Aspergillus* (Table-5). Nevertheless, it was possible to detect two isolates of *Trichoderma* spp. in the ruminal fluid of the SC group and an isolate of *Paecilomyces* spp. in the feces of an animal from the CC group (Table-5).

**Table-5:** Distribution of genera of mycelial fungi isolated from the ruminal fluid and feces of Nellore calves supplemented (SC) or not supplemented (CC) with ruminal fungi.

Genera	Total (n)	Ruminal fluid				Feces			
	
		CC		SC^1^		CC		SC^1^	
			
		n	%	n	%	n	%	n	%
*Aspergillus* spp.	20**	5	25	5	25	6	30	4	20
*Trichoderma* spp.	2	-	-	2	100	-	-	-	-
*Paecillomyces* spp.	1	-	-	-	-	1	100	-	-
Total	23	5		7		7		7	

** Values significantly higher when compared between treatments and between genders using the Chi-square test (p=0.01). ^1^Supplementation with fungi: *Aspergillus terreus* (isolated VN15) and *Trichoderma longibrachiatum* (isolated VN20)

### Quantification and identification of genera of rumen protozoa

The SC group did not differ (p>0.05) in the total concentration or in the subpopulations of the protozoa (small, medium, and large) in the ruminal fluid, when compared to the CC group (Table-6) [21]. In this study, 11.246 ruminal fluid ciliates were identified and in the analysis of the distribution profile of the *Eodinium* spp., this genus was detected only in SC (p<0.05 – Table-6).

**Table-6:** Mean concentrations, standard deviations (SD), and distribution of protozoan sizes and genera in the ruminal fluid of Nellore calves supplemented (SC) or not supplemented (CC) with ruminal fungi.

Variables	CC		SC^1^		p-value
	
	n (/mL)	%	n (/mL)	%	
Classification by size					
Small^2^	50.3±61.8	7.0	41.7±33.2	6.1	0.75
Medium^2^	624.6±163.6	87.2	582.5±216.6	84.5	0.40
Large^2^	41.8±47.2	5.8	64.9±60.9	9.4	0.37
Genus quantification					
*Buetschlia* spp.	34.9±55.1	4.9	24.0±30.3	3.5	0.74
*Isotricha* spp.	67.5±53.2	9.4	86.5±35.2	12.6	0.26
*Dasytricha* spp.	37.0±39.7	5.2	65.7±48.3	9.5	0.26
*Charonina* spp.	15.4±32.2	2.1	17.7±13.1	2.6	0.21
*Entodinium* spp.	440.5±136.8	61.3	370.6±203.5	53.8	0.24
*Diplodinium* spp.	28.6±29.9	4.0	24.6±12.0	3.0	0.91
*Eodinium* spp.	0.00±0.0	0.0	6.9±11.2	1.0	*0.02
*Eremoplaston* spp.	1.1±2.2	0.2	1.6±4.6	0.2	0.64
*Eudiplodinium* spp.	14.7±15.7	2.1	24.9±31.5	3.6	0.70
*Diploplastron* spp.	27.6±29.4	3.9	16.2±13.8	2.4	0.70
*Polyplastron* spp.	13.1±18.3	1.8	4.9±6.9	0.7	0.45
*Ostracodinium* spp.	11.6±16.9	1.6	3.7±5.8	0.5	0.58
*Elytroplastron* spp.	7.0±12.6	1.0	3.1±5.9	0.5	0.52
*Metadinium* spp.	13.5±16.9	1.9	14.5±11.5	2.1	0.55
*Enoploplastron* spp.	0.4±1.1	0.1	1.2±3.5	0.2	0.92
*Ophyroscolex* spp.	0.4±1.1	0.1	20.6±44.9	3.0	0.19
*Epidinium* spp.	4.4±8.2	0.6	3.7±10.6	0.5	0.64
Total	716.6±141.4	100.0	689.1±254.3	100.0	0.67

Test of comparison of means by the Kruskal–Wallis test with 5% significance. ^1^Supplementation with fungi: *Aspergillus terreus* (isolated VN15) and *Trichoderma longibrachiatum* (isolated VN20). ^2^Small (up to 40×60 μm) medium (up to 100×150 μm) and large (>100×150 μm) [21]

## Discussion

### Analysis of performance, physicochemical characteristics, and macroscopic characteristics of ruminal fluid

In this study, the supplementation with the two facultative anaerobic fungi in the rumen did not significantly change the animals’ performance, despite promoting a numerical increase of 2.63 kg in the average total gain. Different factors, such as the type of microorganism used and its dosage, the nature of the diet, the physiological status of the animal, and the feeding strategy [28,29], could be attributed to the fungi not having an influence on the performance of the calves in the present study. In addition, the animals in this study were still receiving balanced diets and thus, the fungi did not contribute to improving the performance of those calves that received the lignified forage. Despite the low protein value of hay, the total diet met the protein requirements for growing cattle with an average body weight of 280.0 kg and daily weight gain of 0.400 kg/day, according to the recommendations of the NRC [16]. The low weight gains observed in the present study were compatible with those detected in animals grazing on tropical forages in the dry season of semiarid regions. However, future studies should evaluate diets with increased protein value to favor the improved growth of inoculated fungi and, consequently, their expression of cellulolytic enzymes.

Another study also observed that the DWG (1.47 kg/day) was not influenced by the addition of amylase enzymes from *Aspergillus awamori* in Nellore bulls finished in confinement, who received Tifton hay as bulky hay [30]. However, Aguirre *et al*. [31] observed that newly weaned Pelibuey sheep, fed with Buffel grass (*Cenchrus ciliaris* L.), and supplemented with exogenous enzymes from *T. longibrachiatum*, showed improvements in DWG and TWG, in relation to the non-supplemented group.

Supplementation did not alter the patterns of the ruminal fluid’s physical characteristics and the PRAM, which were in the normal range according to Dirksen [21]. The pH considered normal for ruminal fluid is in the range of 5.5-7.6, and is vital for the survival and stability of rumen cellulolytic microorganisms [21]. The pH of the ruminal fluid in the calves was also within the normal range. However, supplementation reduced pH values by 3.76% for SC when compared to CC. This result could indicate greater levels of microbial activity for animals that were fed with fibrous diets and evaluated under fasting [21].

In other studies, the addition of fungi and/or fungal enzymes to the ruminant diet improved the microbiota, stabilized rumen pH, promoted microbial degradation of the plant cell wall, and, consequently, helped to improved animal performance [32,33].

### Quantification and identification of Enterobacteriaceae genera

The inclusion of ruminal fungi did not alter the Enterobacteriaceae populations, which presented values close to those of other studies that evaluated calves fed on pasture without supplementation [34]. In another study, after 60 days of experimentation, Stellaa *et al*. [35] also showed supplementation with a commercial strain of *Saccharomyces cerevisiae* (CNCM I-1077), in goats of the Saanen breed that were fed with triticale silage (×*Triticosecale* Wittmack), did not alter the fecal elimination concentrations of these bacteria when compared to the animals in the control group.

Considering the genus profile of Enterobacteriaceae, *Escherichia* spp. and *Enterobacter* spp. were the most identified genera in this study. When evaluating the effect of *S. cerevisiae* supplementation on the diet of crossbred Friesian×Baladi calves, during the transition period from milk to berseem hay (*Trifolium alexandrinum*), Hassan *et al*. [36] reported results similar to the present study with *Escherichia* spp. being the most identified.

### Quantification and identification of the genera of facultative anaerobic fungi

In this study, the average concentration of facultative mycelial anaerobic fungi and yeasts in the ruminal fluid and in the feces was not significantly influenced by supplementation. However, the detected concentrations were higher than those described by Abrão *et al*. [9], who detected an average of 3.3×10³ CFU/mL in samples of ruminal fluid from Nellore calves, fed exclusively in tropical pastures of low nutritional value and without supplementation, in northern Minas Gerais, Brazil. This higher concentration could be related to the diet containing the highest grain content for the animals evaluated in the present study.

The genus of mycelial fungi that was most frequently identified in the rumen fluid and in the feces of the calves was *Aspergillus*. It was also possible to identify two isolates of *Trichoderma* spp., in ruminal fluid from SC that had morphological characteristics similar to one of the fungi used in the supplementation, indicating that this fungus could remain viable in the rumen environment.

The *Aspergillus* genus was also the most identified in samples from Nellore calves, fed on pastures of *Urochloa* spp. without supplementation during the dry season [9]. The predominance of this genus could be justified by its versatility and efficiency in the metabolization of different carbon sources, including lignified cellulose [11,37].

### Quantification and identification of genera of rumen protozoa

In this study, supplementation with facultative anaerobic fungi did not influence the average concentration of rumen protozoa. There is a study performed with Nellore calves of both sexes, with the same age group and a feeding regimen similar to the one of this study, but without supplementation with fungi. In that study, Duarte *et al*. [38] detected a higher population of protozoa (1.7±9.1×10^4^/mL) in the ruminal fluid. The lower concentration of ciliates observed in the calves evaluated in this study could be justified by the nutritional conditions of the hay used, which had low protein levels and high proportions of lignin, in addition to the fact that the animals remained in isolated stalls, which could have influenced colonization.

In total, 17 genera of protozoa were identified in this study. As described by Wright [39], the genera *Entodinium*, *Isotricha*, and *Dasytricha* were the most frequently found in the evaluated calves. In another study, Duarte *et al*. [38] detected *Buetschilia* as the genus of ruminal ciliates most identified in Nellore calves that were raised in tropical pastures during the dry season, without supplementation. The diversity of the genera of these eukaryotes may indicate a healthy ruminal environment [40], as even in the dry season, it was possible to observe this in our study.

Among the protozoa identified, *Eodinium* spp. was detected only in SC. This genus of protozoa is a member of the Ophryoscolecidae family, which is preferably characterized as cellulolytic [41] and considered important for the degradation of cellulose present in the forage used. Silva *et al*. [40] and Nigri *et al*. [42] also included the genus *Eodinium* among those most frequently found in Nellore steers fed with *Urochloa* spp. hay, without supplementation.

## Conclusion

The supplementation with the two selected isolates of *A. terreus* and *T. longibrachiatum* has potential for use as possible additives. This is because they did not alter the physiological parameters of the composition of the facultative anaerobic microbiota of the rumen and feces. Furthermore, they also favor the presence of the protozoa, *Eodinium* spp. However, future studies should be performed to better define the dosages and strategies for use in supplementing the diet of calves.

## Authors’ Contributions

ASC and ERD: Designed the study and drafted and revised the manuscript. LMGF, PPXC, and VSMJ: Managed the animals. SAF: Participated in assembling the microorganism strains and culture conditions. TAXS: Participated in the assembling of the microorganism strains and culture conditions and wrote and revised the paper. All authors have read and approved the final manuscript.
